# Evaluation of research productivity among academic neuro-ophthalmologists using the relative citation ratio

**DOI:** 10.3389/fopht.2026.1813075

**Published:** 2026-08-06

**Authors:** Matthew N. Henderson, Lucy S. Guan, Aidan Gilson, Devon A. Cohen

**Affiliations:** 1 Cole Eye Institute, Cleveland Clinic Foundation, Cleveland, OH, United States; 2 Department of Ophthalmology, Rutgers Robert Wood Johnson Medical School, New Brunswick, NJ, United States; 3 Department of Ophthalmology, Lewis Katz School of Medicine, Temple University, Philadelphia, PA, United States; 4 Department of Ophthalmology, Harvard Medical School, Massachusetts Eye and Ear, Boston, MA, United States

**Keywords:** academia, academic medicine, bibliometrics, neuro-ophthalmology, relative citation ratio, research productivity

## Abstract

**Background:**

Bibliometric analyses within the field of neuro-ophthalmology are limited. The current study utilizes the relative citation ratio (RCR), a National Institutes of Health (NIH) sponsored index that separates measures of research impact and productivity into independent variables, to evaluate scholarly output within this cohort.

**Methods:**

Fellowship-trained neuro-ophthalmologists at Accreditation Council for Graduate Medical Education (ACGME)-accredited institutions in the United States were indexed using the NIH iCite database. Publication count, mean RCR score (mRCR), and weighted RCR score (wRCR) were obtained between October 2023 and January 2024. Data were compared using univariate and multivariate ordinary least squares regression analyses, and chi-square tests were used to compare categorical variables.

**Results:**

The cohort included 256 neuro-ophthalmologists from 103 institutions. The majority were male (60.9%), ophthalmology trained (69.1%), and did not hold a PhD (88.3%). Median publication count was 26 (interquartile range [IQR], 8.25–84.50), median mRCR was 1.23 (IQR, 0.78–1.68), and median wRCR was 31.48 (IQR, 7.61–99.94). Professor rank was independently associated with significantly higher publication counts, mRCR, and weighted RCR. Completion of a neurology residency was independently associated with higher mean RCR but not publication count or weighted RCR. Gender was not independently associated with publication count, mRCR, or wRCR after multivariable adjustment. Female faculty were significantly underrepresented among professors despite greater representation within more recent residency cohorts.

**Conclusion:**

Academic neuro-ophthalmologists demonstrate high levels of research productivity and impact, with a median mRCR exceeding the NIH benchmark value of 1.0. Academic rank was the strongest independent predictor of cumulative scholarly productivity and article level influence, while completion of a neurology residency was associated with higher average article influence but not greater overall scholarly output. Although research productivity was comparable after adjustment for career stage and academic rank, women remain underrepresented in senior academic positions. These findings establish benchmark RCR values for academic neuro-ophthalmology and provide a framework for future investigations of scholarly development and academic advancement.

## 1 Introduction

Measures of research productivity have important implications for decisions regarding faculty hiring, promotion, and allocation of research funding (1, 2). While commonly utilized bibliometrics have attempted to objectively and accurately gauge research output, many have been criticized as an oversimplification of scholarly activity, often conflating various independent measures into a single value (3, 4). Numerous metrics, such as the h-index, are heavily influenced by the career duration, therefore disadvantaging younger researchers or those who have experienced career interruptions, such as women taking breaks for parental leave (3). Furthermore, many suffer from a lack of field standardization, which hinders the comparability of authors working across different specialties of varying sizes and scopes (4–6).

In light of these shortcomings, the National Institutes of Health (NIH) introduced the relative citation ratio (RCR), an article-level metric aimed to better quantify research impact and productivity (7). The RCR is determined by dividing an article’s citation rate (total citations per year) by the expected citation rate, which is based on the performance of co-cited articles benchmarked against NIH R01-funded publications. This allows for standardization of an article’s impact relative to others in its field, with an average NIH RCR value of 1. Unique to the RCR is its incorporation of a co-citation network, which facilitates field standardization and thus enables direct comparisons of impact across publications at both inter- and intra-field levels (7, 8). This metric has been validated in a dataset of over 88,000 publications, demonstrating consistency with expert opinion of research quality (7).

Mean and weighted RCR values are calculated by averaging and summing, respectively, all article-level RCR scores for an individual (7). The mean RCR (mRCR) assesses an author’s overall impact within their field, independent of time-dependent factors such as total publication or citation counts. The weighted RCR (wRCR) considers an author’s total productivity by factoring in number of publications. By separating measures of impact and productivity into distinct metrics, the RCR provides a unique opportunity for a more nuanced approach to bibliometric analyses.

To date, no bibliometric analyses have been conducted among academic neuro-ophthalmologists. Application of the RCR to other academic ophthalmologic subspecialties has provided insight into gender, career length, and academic rank differences in research productivity, however, the field of neuro-ophthalmology lacks similar investigation (9–12). Given the absence of prior bibliometric analyses in academic neuro-ophthalmology, this study was designed as an exploratory analysis to characterize research impact and productivity using the RCR and evaluate associations between faculty characteristics and bibliometric measures.

## 2 Materials and methods

### 2.1 Inclusion and exclusion criteria

A list of all ophthalmology residency programs accredited by the Accreditation Council for Graduate Medical Education (ACGME) in the United States was accessed in May 2022 (https://apps.acgme.org/ads/public/). The department websites of all 125 ACGME-accredited programs were searched to identify faculty members who completed a neuro-ophthalmology fellowship following ophthalmology or neurology residency. A total of 103 ophthalmology programs were found to have at least one fellowship-trained neuro-ophthalmologist. All listed faculty that met the criteria were included. Faculty demographics, including gender, residency type, acquisition of a Doctor of Philosophy (PhD), academic rank, and career duration, were collected using department websites, Doximity, and other publicly available professional sources. When not explicitly stated, gender was assigned only when there was consistent identification across institutional and professional sources; no faculty members required exclusion because gender could not be determined. Academic ranks included assistant professor, associate professor, professor, and “other”, which included titles with qualifiers, such as “clinical” or “adjunct”; titles such as “instructor”, “lecturer”, or “staff”; and otherwise, unspecified titles. Career duration was determined by using residency start year and further stratified faculty into the following categories: ≤1980, 1981-1990, 1991-2000, 2001-2010, >2010. For faculty members who obtained a medical degree internationally, residency start year was defined as the beginning of their first neurology or ophthalmology residency. The study was deemed exempt from Institutional Review Board review.

### 2.2 Bibliometric analysis

The NIH iCite Website (https://icite.od.nih.gov/) was accessed between October 2023 and January 2024 to collect publication counts, mRCR, and wRCR for each neuro-ophthalmologist. Only PubMed-indexed publications and citations starting in 1980 are included in the iCite database. Before 2002, authors’ first and middle names are represented as initials in PubMed. Thus, faculty members who began their residency before 2002 were searched using first and middle initials, if applicable, along with their last name. Those who began residency training in 2002 or later were searched using their full first and last names. For faculty with hyphenated or parenthesized last names, both names and the full hyphenated name, if applicable, were utilized for indexing. Publications were then consolidated in these instances to yield aggregate values. This occurred for 7 faculty (2.7%). Individual publications were reviewed with attention to ophthalmology, neurology, and basic science adjacent journals to remove those from authors of similar names from other medical specialties or scientific fields. For authors with names generating exceedingly high numbers of publications that could not feasible be reviewed manually, publicly available sources, including departmental websites, ResearchGate, ORCiD, and Doximity, were used for cross-referencing publications.

### 2.3 Statistical analysis

Data were compared by gender, career duration, academic rank, residency type, and acquisition of a PhD. Due to the skewed distribution of data, the results are presented as the median and interquartile ranges (IQR). Univariate and multivariate ordinary least squares regression were used for subgroup analyses of influencing publication count, mRCR, and wRCR, including gender, type of residency completed, PhD status, academic rank, and career duration. The significance threshold was set at 0.05. To account for potential heteroskedasticity, all linear regressions were estimated using heteroskedasticity-consistent standard errors (HC3). Chi-squared goodness-of-fit test was used to determine whether proportions of academic title and residency start year differed between genders. Statistical analyses were performed using IBM SPSS Statistics for Windows, version 27 (IBM Corp., Armonk, N.Y., USA).

## 3 Results

The analysis included a cohort of 256 academic neuro-ophthalmologists from 102 institutions (**Table 1**). The median number of publications was 26 (IQR, 8.25-84.50) (**Table 2**), the median mRCR was 1.23 (IQR, 0.78 – 1.68) (**Table 3**), and the median wRCR was 31.48 (IQR, 7.61 – 99.94) (**Table 4**).

**Table 1:** Demographics of neuro-ophthalmology faculty (n = 256).

Characteristic	Number	Percent
Gender		
Female	100	39.1
Male	156	60.9
Residency		
Ophthalmology	177	69.1
Neurology	68	26.6
Both	11	4.3
PhD degree		
No	226	88.3
Yes	30	11.7
Academic rank		
Other^*^	71	27.7
Assistant Professor	64	25.0
Associate Professor	47	18.4
Professor	74	28.9
Residency start year		
≤1980	37	14.5
1981 - 1990	49	19.1
1991 - 2000	51	19.9
2001 - 2010	62	24.2
>2010	57	22.3

^*^ Other includes instructors, lecturers, titles with qualifiers such as “clinical”, “adjunct”, or “affiliate”, and faculty not otherwise specified.

**Table 2:** Publications by gender, residency, PhD acquisition, academic ranking, and residency start year.

Characteristic	Median (IQR)	Univariate		Multivariate	
		Coefficient (95% CI)	*P* value^*^	Coefficient (95% CI)	*P* value^*^
Gender					
Female	17.5 (8.0 to 41.5)	Reference		Reference	
Male	34.0 (11.0 to 109.0)	40.8 (19.6 to 62.1)	**0.0002**	12.3 (-6.6 to 31.1)	0.20
Residency					
Neurology	18.5 (8.3 to 60.0)	Reference		Reference	
Ophthalmology	27.0 (8.5 to 91.5)	9.1 (-17.8 to 35.9)	0.51	-0.5 (-25.0 to 24.1)	0.97
Both	64.0 (8.0 to 171.0)	55.9 (-28.7 to 140.5)	0.20	22.3 (-54.0 to 98.6)	0.57
PhD degree					
No	23.0 (8.0 to 81.3)	Reference		Reference	
Yes	61.5 (16.0 to 138.5)	29.1 (-5.9 to 64.0)	0.10	17.0 (-10.9 to 44.9)	0.23
Academic rank					
Other^†^	11.0 (5.0 to 26.0)	8.3 (-2.5 to 19.8)	0.13	0.1 (-12.2 to 12.3)	0.99
Assistant Professor	10.5 (4.0 to 24.0)	Reference		Reference	
Associate Professor	33.0 (16.0 to 78.0)	31.8 (18.1 to 45.5)	**<0.0001**	29.9 (12.8 to 47.0)	**0.0006**
Professor	110.0 (57.0 to 178.3)	128.6 (97.6 to 159.6)	**<0.0001**	112.1 (79.5 to 144.7)	**<0.0001**
Residency start year					
≤1980	82.0 (22.5 to 142.0)	Reference		Reference	
1981 - 1990	33.0 (12.0 to 118.5)	-13.2 (-62.1 to 35.8)	0.60	-4.8 (-50.2 to 40.6)	0.84
1991 - 2000	58.0 (23.0 to 123.0)	-11.1 (-59.6 to 37.4)	0.65	-9.6 (-58.9 to 39.7)	0.70
2001 - 2010	16.0 (6.8 to 38.5)	-69.9 (-106.5 to -33.4)	**0.0002**	-21.7 (-60.3 to 16.8)	0.27
>2010	10.0 (4.0 to 23.0)	-87.8 (-122.8 to -52.6)	**<0.0001**	-15.8 (-53.6 to 21.9)	0.41
Total	26.0 (8.3 to 84.5)				

^*^ P values generated using univariate and multivariate ordinary least squares regression. Bold values indicate statistical significance at the <0.05 significance level.

^†^ Other includes instructors, lecturers, titles with qualifiers such as “clinical”, “adjunct”, or “affiliate”, and faculty not otherwise specified.

**Table 3:** Mean RCR by gender, residency, PhD acquisition, academic ranking, and residency start year.

Characteristic	Median (IQR)	Univariate		Multivariate	
		Coefficient (95% CI)	*P* value^*^	Coefficient (95% CI)	*P* value^*^
Gender					
Female	1.30 (0.78 to 1.56)	Reference		Reference	
Male	1.18 (0.80 to 1.87)	0.23 (-0.02 to 0.48)	0.07	0.06 (-0.22 to 0.35)	0.65
Residency					
Neurology	1.36 (0.80 to 2.00)	Reference		Reference	
Ophthalmology	1.15 0.77 to 1.63)	-0.52 (-0.98 to -0.07)	**0.025**	-0.58 (-1.07 to -0.10)	**0.018**
Both	1.56 (1.28 to 1.92)	-0.32 (-0.89 to 0.25)	0.28	-0.54 (-1.27 to 0.19)	0.15
PhD degree					
No	1.15 (0.77 to 1.63)	Reference		Reference	
Yes	1.51 1.28 to 1.90)	0.28 (-0.05 to 0.62)	0.10	0.26 (-0.07 to 0.59)	0.13
Academic rank					
Other^†^	1.10 (0.78 to 1.74)	0.13 (-0.22 to 0.48)	0.47	0.15 (-0.28 to 0.57)	0.50
Assistant Professor	1.13 (0.61 to 1.53)	Reference		Reference	
Associate Professor	1.14 (0.76 to 1.62)	0.07 (-0.27 to 0.41)	0.70	0.10 (-0.31 to 0.51)	0.64
Professor	1.48 (1.10 to 2.01)	0.53 (0.12 to 0.95)	**0.011**	0.47 (0.02 to 0.91)	**0.040**
Residency start year					
≤1980	1.37 (1.10 to 1.90)	Reference		Reference	
1981 - 1990	1.20 (0.87 to 1.55)	-0.56 (-1.19 to 0.07)	0.08	-0.58 (-1.22 to 0.06)	0.08
1991 - 2000	1.43 (0.87 to 2.27)	-0.15 (-0.83 to 0.53)	0.66	-0.18 (-0.91 to 0.55)	0.63
2001 - 2010	1.18 (0.65 to 1.64)	-0.58 (-1.22 to 0.05)	0.07	-0.46 (-1.15 to 0.22)	0.18
>2010	1.10 (0.64 to 1.55)	-0.48 (-1.17 to 0.21)	0.18	-0.36 (-1.13 to 0.42)	0.36
Total	1.23 (0.78 to 1.68)				

^*^ P values generated using univariate and multivariate ordinary least squares regression. Bold values indicate statistical significance at the <0.05 significance level.

^†^ Other includes instructors, lecturers, titles with qualifiers such as “clinical”, “adjunct”, or “affiliate”, and faculty not otherwise specified.

**Table 4:** Weighted RCR by gender, residency, PhD acquisition, academic ranking, and residency start year.

Characteristic	Median (IQR)	Univariate		Multivariate	
		Coefficient (95% CI)	*P* value^*^	Coefficient (95% CI)	*P* value^*^
Gender					
Female	15.63 (7.03 to 59.01)	Reference		Reference	
Male	45.93 (9.90 to 158.90)	75.10 (29.44 to 120.76)	**0.0012**	21.84 (-21.44 to 65.12)	0.32
Residency					
Neurology	22.66 (6.64 to 96.82)	Reference		Reference	
Ophthalmology	30.60 (7.90 to 99.34)	-4.80 (-64.09-54.49)	0.87	-22.78 (-77.33 to 31.76)	0.41
Both	99.38 (12.61 to 308.00)	79.25 (-73.40 to 231.90)	0.31	15.87 (-122.31 to 154.05)	0.82
PhD degree					
No	25.16 (7.03 to 96.08)	Reference		Reference	
Yes	67.38 (20.83 to 224.30)	49.98 (-18.37 to 118.32)	0.15	27.08 (-34.15 to 88.31)	0.39
Academic rank					
Other^†^	11.60 (5.77 to 33.05)	18.94 (1.37 to 36.51)	**0.035**	8.62 (-12.48 to 29.72)	0.42
Assistant Professor	9.16 (4.61 to 22.58)	Reference		Reference	
Associate Professor	43.68 (9.64 to 90.40)	48.00 (27.31 to 68.69)	**<0.0001**	37.04 (5.41 to 68.67)	**0.022**
Professor	141.10 (79.07 to 328.30)	234.44 (159.31 to 309.57)	**<0.0001**	205.87 (128.71 to 283.04)	**<0.0001**
Residency start year					
≤1980	102.60 (23.17 to 260.00)	Reference		Reference	
1981 - 1990	51.64 (10.31 to 164.50)	-43.83 (-129.07 to 41.41)	0.31	-29.16 (-109.80 to 51.47)	0.48
1991 - 2000	77.43 (32.17 to 147.60)	-2.78 (-117.86 to 112.30)	0.96	4.10 (-119.29 to 127.48)	0.95
2001 - 2010	14.34 (6.41 to 69.86)	-126.04 (-190.17 to -61.92)	**0.0001**	-32.78 (-111.68 to 46.13)	0.42
>2010	7.68 (4.86 to 19.26)	-157.95 (-219.33 to -96.57)	**<0.0001**	-33.14 (-112.74 to 46.46)	0.41
Total	31.48 (7.61 to 99.94)				

^*^ P values generated using univariate and multivariate ordinary least squares regression. Bold values indicate statistical significance at the <0.05 significance level.

^†^ Other includes instructors, lecturers, titles with qualifiers such as “clinical”, “adjunct”, or “affiliate”, and faculty not otherwise specified.

### 3.1 Gender

The study cohort was predominantly male (60.9%). Male faculty exhibited greater publication counts (17.5 [IQR, 8.0-41.5] vs. 34.0 [IQR, 11.0-109.0]; p = 0.0002) and wRCR scores compared to females (15.63 [IQR 7.03-59.01] vs. 45.93 [IQR 9.90-158.90]; p = 0.012). No difference in mRCR score was observed between genders (1.30 [IQR 0.78 to 1.56] vs. 1.18 [IQR 0.80 to 1.87]; p = 0.07). Multivariate analysis did not demonstrate a significant independent influence of gender on publication count (12.3 [-6.6 to 31.1], p = 0.20 [relative to female]), mRCR (0.06 [-0.22 to 0.35], p = 0.65 [relative to female]), or wRCR (21.84 [-21.44 to 65.12], p = 0.32 [relative to female]).

**Figure 1:**
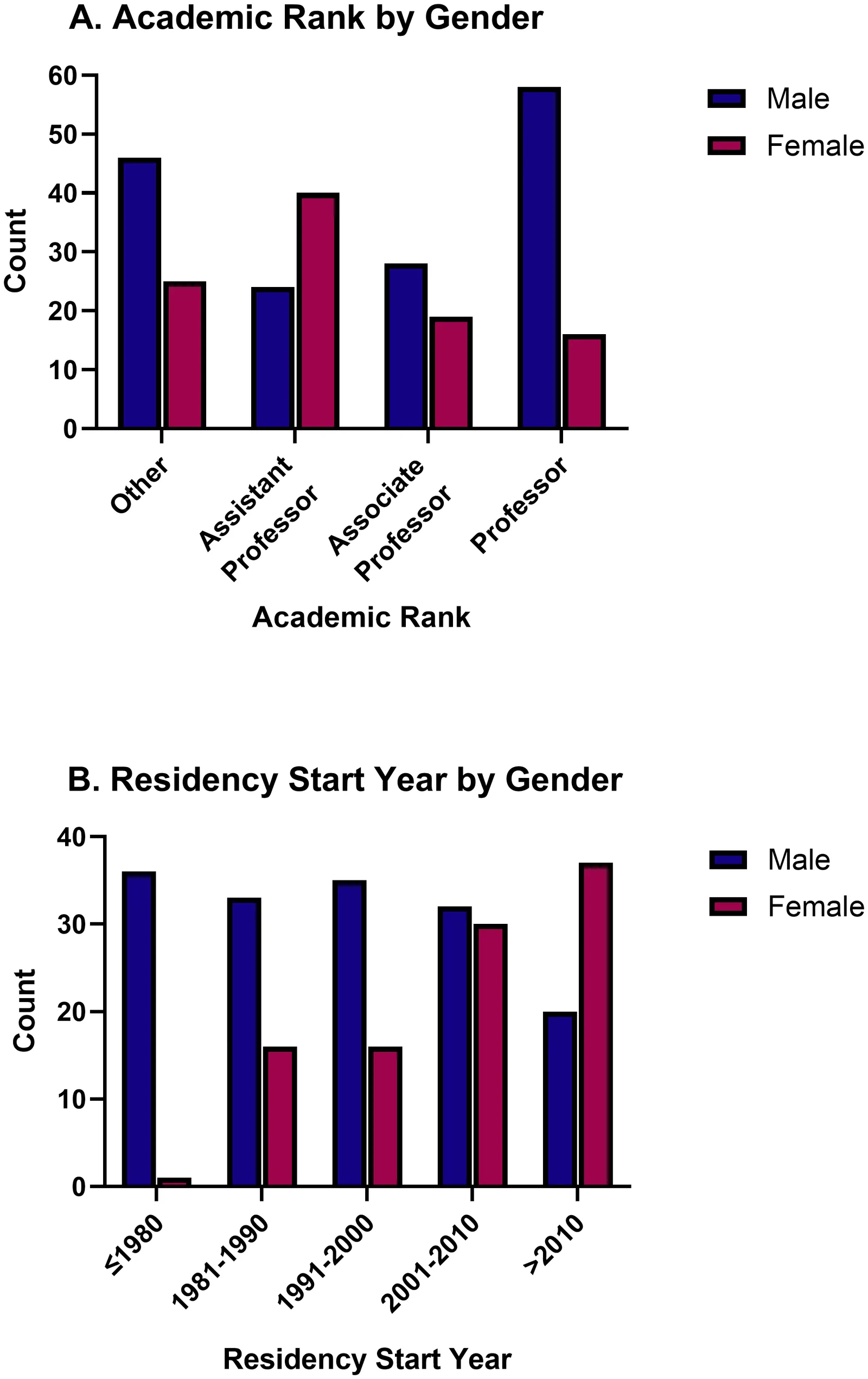
Histogram of academic rank **(A)** and residency start year **(B)** distribution by gender demonstrating underrepresentation of female gender in the academic rank of professor and earlier residency start dates.

The distribution of academic titles significantly varied by gender (*X^2^* [3, N = 256] = 24.70, P < 0.0001), with female faculty being underrepresented in higher academic ranks (**Figure 1A**). The distribution of residency start year also differed significantly by gender (*X^2^* [4, N = 256] = 40.93, p < 0.0001) with fewer female faculty starting their careers in earlier decades (**Figure 1B**).

### 3.2 Residency type

The majority of neuro-ophthalmology faculty completed an ophthalmology residency (69.1%) compared to those who completed a neurology residency (26.6%) or both ophthalmology and neurology residencies (4.3%) (**Table 1**). Univariate and multivariate analysis demonstrated no statistical differences in publication number and wRCR when comparing type of residency completed. However, completion of a neurology residency was associated with higher mRCR than completion of an ophthalmology residency on univariate (-0.52 [-0.98 to -0.07], p = 0.025 [relative to neurology]) and multivariate analysis (-0.58 [-1.07 to -0.10], p = 0.018 [relative to neurology]).

### 3.3 PhD acquisition

30 (11.7%) faculty members held a PhD degree. No significant differences in publication count, mRCR or wRCR were demonstrated on univariate or multivariate analyses in faculty holding a PhD relative to those without a PhD.

### 3.4 Academic rank

The most prevalent academic rank in this cohort was professor (28.9%), followed by “other” (27.7%), assistant professors (25%), and associate professors (18.4%). The academic title of professor was associated with greater publication counts (p < 0.0001), mRCR (0.040), and wRCR (p < 0.0001) relative to the reference of assistant professor on both univariate and multivariate analyses. The academic title of associate professor was associated with greater publication counts (p = 0.0006) and wRCR (p = 0.022) relative to the reference of assistant professor on both univariate and multivariate analyses.

### 3.5 Career duration

The greatest number of faculty started their residency between 2001-2010 (24.2%), followed by >2010 (22.3%), 1991-2000 (19.9%), 1981-1990 (19.1%), and ≤1980 (14.5%). Faculty starting residency between 2001 and 2010 (p = 0.0002) and after 2010 (p < 0.0001) demonstrated significantly fewer publications relative to those starting their careers on or before 1980 on univariate analysis, however, no significant differences were observed on multivariate analysis. No significant difference in mRCR was observed between cohort of career start dates on univariate or multivariate analyses. Faculty starting residency between 2001 and 2010 (p = 0.0001) and after 2010 (p < 0.0001) demonstrated significantly lower wRCR relative to those starting their careers on or before 1980 on univariate analysis. No significant differences were observed on multivariate analysis.

## 4 Discussion

To our knowledge, this is the first bibliometric analysis of academic neuro-ophthalmologists using the National Institutes of Health (NIH) Relative Citation Ratio (RCR). By separately evaluating publication count, mean RCR (mRCR), and weighted RCR (wRCR), this study provides a comprehensive assessment of both research productivity and article-level influence within the academic neuro-ophthalmology workforce. These findings establish benchmark bibliometric values for the specialty and provide a foundation for future investigations of academic scholarship, faculty development, and workforce trends.

The distinction between mRCR and wRCR is an important feature of the RCR methodology. Mean RCR reflects the average influence of an author’s publications independent of publication volume, whereas weighted RCR incorporates both article influence and scholarly output, representing cumulative research impact. Accordingly, these metrics should be interpreted as complementary rather than interchangeable measures of research performance. Across the cohort, academic neuro-ophthalmologists demonstrated a median mRCR of 1.23, exceeding the NIH benchmark value of 1.0 and indicating that, on average, publications authored by neuro-ophthalmologists receive greater citation influence than the typical R01 NIH-funded publication. Simultaneously, the median weighted RCR and publication count demonstrate substantial cumulative scholarly productivity across the specialty.

Among the variables examined, academic rank demonstrated the strongest and most consistent association with research productivity. Professors exhibited significantly greater publication counts, mean RCR, and weighted RCR than junior faculty, even after adjustment for career duration and other covariates. These findings are consistent with previous bibliometric studies across ophthalmology and other medical specialties and likely reflect the cumulative nature of scholarly activity, the importance of sustained research productivity in academic promotion, and increased opportunities for mentorship, collaboration, and research leadership that accompany advancement through academic ranks (2, 13–17).

Completion of a neurology residency was independently associated with higher mean RCR, whereas publication count and weighted RCR did not differ significantly by residency background. Because weighted RCR incorporates publication volume and represents cumulative research influence and, these findings should not be interpreted as evidence that neurology-trained faculty have greater overall scholarly impact. Rather, the observed association suggests differences in the average citation influence of individual publications, the underlying reasons for which remain uncertain. Future investigations evaluating research focus, publication type, funding mechanisms, collaborative networks, and institutional characteristics may help clarify this relationship. We have intentionally avoided attributing this finding to differences in fellowship training or clinical practice patterns, as these factors were not evaluated in the present study.

Although faculty were identified through ACGME-accredited ophthalmology residency programs, the residency training distribution within our cohort closely parallels that reported in a recent national demographic study of United States neuro-ophthalmologists, which included members of the American Academy of Ophthalmology, North American Neuro-Ophthalmology Society, American Neurological Association, and American Academy of Neurology (18). That study reported that 67.6% of neuro-ophthalmologists completed ophthalmology residency, 25.0% completed neurology residency, and 7.4% completed both, compared with 69.1%, 26.6%, and 4.3%, respectively, in our cohort. The close agreement between these independent datasets supports the representativeness of our study population despite identification through ophthalmology residency programs. Nevertheless, future bibliometric analyses incorporating neurology residency programs may provide an even more comprehensive characterization of the academic neuro-ophthalmology workforce.

Gender differences were similarly clarified after adjustment for potential confounders. While male faculty demonstrated greater publication counts and weighted RCR on univariate analyses, these associations were no longer significant following multivariable adjustment, suggesting that differences in scholarly productivity were largely explained by other factors, including academic rank and career duration, rather than gender itself. The significant differences in academic rank and residency cohort distributions observed between males and females (**Figure 1**) further support the interpretation that career stage, rather than gender itself, largely explains the unadjusted differences in scholarly productivity. At the same time, women remained substantially underrepresented among professors, with only 21.6% of faculty holding the rank of professor being female. Because this cross-sectional study cannot determine the mechanisms underlying this disparity, our findings should not be interpreted as evidence of inequities within the promotion process alone. Rather, the observed distribution may reflect multiple contributing factors, including historical workforce demographics, differences in career stage, institutional promotion practices, mentorship opportunities, and other structural influences that warrant further investigation. Encouragingly, female representation increased substantially among more recent residency cohorts, suggesting continued progress toward a more balanced academic workforce.

The present findings have practical implications for academic departments and faculty development. Objective bibliometric measures are increasingly incorporated into decisions regarding recruitment, promotion, mentorship, and research funding. By establishing specialty-specific benchmark values for publication count, mean RCR, and weighted RCR, this study provides a contemporary reference against which academic neuro-ophthalmologists may compare scholarly activity while recognizing that bibliometric measures represent only one component of academic success. Clinical excellence, education, mentorship, administrative leadership, and service remain essential contributions that are not captured by citation-based metrics.

Several limitations merit consideration. As with all studies utilizing the NIH iCite database, only PubMed-indexed publications published from 1980 onward are included, potentially underestimating scholarly output among investigators with earlier careers start dates. The RCR does not account for publication type, authorship position, or individual contribution to multi-author publications. Review articles, consensus statements, and large multicenter clinical trials may achieve substantially different citation patterns than original investigations or case reports despite representing different forms of scholarly contribution. Despite careful manual review of publications and consolidation of authors with hyphenated or parenthesized surnames, errors related to author name ambiguity or undocumented name changes remain possible. In these instances, articles were reviewed on a case-by-case basis and cross-referenced with available resources, however, the opportunity for error remains. As women are more likely to undergo a name change during their career, this may result in a disproportionate underestimation of their research productivity.

In conclusion, academic neuro-ophthalmologists demonstrate high levels of scholarly productivity and article-level research impact. Academic rank was the strongest independent predictor of cumulative scholarly output, while completion of a neurology residency was associated with higher mean RCR but not greater cumulative research impact. Women demonstrated research productivity comparable to men after adjustment for career duration and academic rank, although they remain underrepresented in senior academic positions. Together, these findings establish benchmark bibliometric values for academic neuro-ophthalmology and provide a framework for future investigations examining scholarly development, workforce diversity, mentorship, and academic advancement.

## Data Availability

The raw data supporting the conclusions of this article will be made available by the authors, without undue reservation.

## References

[1] Atasoylu AA Wright SM Beasley BW Cofrancesco J Jr Macpherson DS Partridge T . Promotion criteria for clinician-educators. J Gen Intern Med. (2003) 18:711–6. doi: 10.1046/j.1525-1497.2003.10425.x 12950479 PMC1494922

[2] Svider PF Lopez SA Husain Q Bhagat N Eloy JA Langer PD . The association between scholarly impact and National Institutes of Health funding in ophthalmology. Ophthalmology. (2014) 121:423–8. doi: 10.1016/j.ophtha.2013.08.009 24070807

[3] Bornmann L Daniel HD . The state of h index research. Is the h index the ideal way to measure research performance? EMBO Rep. (2009) 10:2–6. doi: 10.1038/embor.2008.233 19079129 PMC2613214

[4] Aoun SG Bendok BR Rahme RJ Dacey RG Batjer HH . Standardizing the evaluation of scientific and academic performance in neurosurgery--critical review of the “h” index and its variants. World Neurosurg. (2013) 80:e85–90. doi: 10.1016/j.wneu.2012.01.052 22381859

[5] Harzing AW Alakangas S . Google Scholar, Scopus and the Web of Science: a longitudinal and cross-disciplinary comparison. Scientometrics. (2016) 106:787–804. doi: 10.1007/s11192-015-1798-9 30311153

[6] Adler R Ewing J Taylor P . Citation statistics. Stat Sci. (2009) 24:1–14. doi: 10.1214/09-STS285 42475790

[7] Hutchins BI Yuan X Anderson JM Santangelo GM . Relative citation ratio (RCR): a new metric that uses citation rates to measure influence at the article level. PloS Biol. (2016) 14:e1002541. doi: 10.1371/journal.pbio.1002541 27599104 PMC5012559

[8] Hutchins BI Baker KL Davis MT Diwersy MA Haque E Harriman RM . The NIH Open Citation Collection: a public access, broad coverage resource. PloS Biol. (2019) 17:e3000385. doi: 10.1371/journal.pbio.3000385 31600197 PMC6786512

[9] Henderson MN Sojitra B Burke O Prenner JL . Evaluation of research productivity among academic vitreoretinal surgeons using the relative citation ratio. Ophthalmol Retina. (2023) 7:509–15. doi: 10.1016/j.oret.2023.01.002 36623728

[10] Guan LS Henderson MN Singh H Guyer O Massaro-Giordano M . Evaluation of research productivity among academic cornea, external diseases, and refractive surgery ophthalmologists using the relative citation ratio. Cornea. (2024) 43(9):1108–14. doi: 10.1097/ICO.0000000000003512 38381040

[11] Henderson MN Singh H Guan L Li A Prenner JL . Evaluation of research productivity among academic glaucoma specialists using the relative citation ratio. Ophthalmol Glaucoma. (2024) 7(6):531–38. doi: 10.1016/j.ogla.2024.06.004 38906253

[12] Henderson MN Guan LS Guyer O Hwang CJ Perry JD . Evaluation of research productivity among academic oculoplastic surgeons using the relative citation ratio. Ophthalmic Plast Reconstr Surg. (2025) 41(6):651–56. doi: 10.1097/IOP.0000000000002940 40131767

[13] Carpenter CR Cone DC Sarli CC . Using publication metrics to highlight academic productivity and research impact. Acad Emerg Med. (2014) 21:1160–72. doi: 10.1111/acem.12482 25308141 PMC4987709

[14] Reddy V Gupta A White MD Gupta R Agarwal P Prabhu AV . Assessment of the NIH-supported relative citation ratio as a measure of research productivity among 1687 academic neurological surgeons. J Neurosurg. (2020) 134(2):638–45. doi: 10.3171/2019.11.JNS192679 32005024

[15] Svider PF Pashkova AA Choudhry Z Agarwal N Kovalerchik O Baredes S . Comparison of scholarly impact among surgical specialties: an examination of 2429 academic surgeons. Laryngoscope. (2013) 123:884–9. doi: 10.1002/lary.23951 23417821

[16] Ponce FA Lozano AM . Academic impact and rankings of American and Canadian neurosurgical departments as assessed using the h index. J Neurosurg. (2010) 113:447–57. doi: 10.3171/2010.3.JNS1032 20380531

[17] Huang G Fang CH Lopez SA Bhagat N Langer PD Eloy JA . Impact of fellowship training on research productivity in academic ophthalmology. J Surg Educ. (2015) 72:410–7. doi: 10.1016/j.jsurg.2014.10.010 25467730

[18] Pakravan P Lai J Cavuoto KM . Demographics, practice analysis, and geographic distribution of neuro-ophthalmologists in the United States in 2023. Ophthalmology. (2024) 131:333–40. doi: 10.1016/j.ophtha.2023.09.020 37739230

